# Valorization of Polypropylene Waste in the Production of New Materials with Adequate Mechanical and Thermal Properties for Environmental Protection

**DOI:** 10.3390/ma15175978

**Published:** 2022-08-29

**Authors:** Maria Râpă, Bogdan Norocel Spurcaciu, Rodica-Mariana Ion, Ramona Marina Grigorescu, Raluca Nicoleta Darie-Niță, Lorena Iancu, Cristian-Andi Nicolae, Augusta Raluca Gabor, Ecaterina Matei, Cristian Predescu

**Affiliations:** 1 Faculty of Materials Science and Engineering, University Politehnica of Bucharest, 313 Splaiul Independentei, 060042 Bucharest, Romania; 2 National Institute for Research & Development in Chemistry and Petrochemistry (ICECHIM), 202 Splaiul Independentei, 060021 Bucharest, Romania; 3 “Petru Poni” Institute of Macromolecular Chemistry, Physical Chemistry of Polymers Department, 41A Grigore Ghica Voda Alley, 700487 Iasi, Romania

**Keywords:** polypropylene waste, thermoplastic elastomer, clay, melt processing, crystallinity, mechanical and thermal properties

## Abstract

Innovative composites based on polypropylene waste impurified cu HDPE (PPW) combined with two thermoplastic block-copolymers, namely styrene-butadiene-styrene (SBSBC) and styrene-isoprene-styrene (SISBC) block-copolymers, and up to 10 wt% nano-clay, were obtained by melt blending. SBSBC and SISBC with almost the same content of polystyrene (30 wt%) were synthesized by anionic sequential polymerization and used as compatibilizers for PPW. Optical microscopy evaluation of the PPW composites showed that the n-clay was encapsulated into the elastomer. Addition of n-clay, together with SBSBC or SISBC, increased the interphase surface of the components in the PPW composites and enhanced the superficial area/volume ratio, which led to a recycled material with improved performance. The data resulting from differential scanning calorimetry (DSC), thermogravimetric analysis (TGA), mechanical evaluation, and dynamic mechanical analysis (DMA) revealed that PPW reinforcement with n-clay and styrene-diene block-copolymers allows the obtaining of composites with favorable mechanical and thermal properties, and excellent impact strength for potential engineering applications.

## 1. Introduction

Today, plastics are used by all of modern society, from injected items to protective equipment for the Coronavirus epidemic, and their increased consumption affects both ecosystems and people’s health. Every year, 5–13 million tons of plastics, accounting for 1.5–4% of global plastics production, end up in the oceans [1]. It has been estimated that from all plastic waste generated, 11% is incinerated and only 19% is recycled [2]. The harmful impact of plastics is related to the occurrence of microplastics, caused by plastic fragmentation, with consequences for the destruction of river, sea, and ocean ecosystems. The effects of microplastic inhalation and ingestion on health are still unknown. The marine plastic crisis has led to challenges to the initial concept applied to plastic manufacturing, from “produce, use, and throw away”, which was characteristic of the linear economy, towards a circular economy based on the closed-loop process of resource–product–renewable resources [3,4]. This challenge will help to reduce the generation of plastic waste, poverty, and gender inequality, and to develop sustainable cities. Examples of the applied circular economy concept are related to food waste [5], plastic packaging [6,7], pyrolysis and gasification [8], the converting of plastic waste into road construction materials [9], etc.

Polypropylene (PP), polyethylene (PE), polystyrene (PS), and polyethylene terephthalate (PET) are the most widespread plastic materials on the market, especially in the packaging field. No later than 31 December 2025, 50% by weight of all packaging plastic waste must be recycled. Globally, PP is the second most versatile plastic [10,11] and is used in the textile industry, construction, packaging, and medical and electrical appliances [10,12,13,14,15], leading to huge waste after their shelf-life has ended. In Europe, plastic waste should be recycled either by mechanical or pyrolysis recycling technologies, as an alternative to incineration and landfilling methods [16]. The recycling and reuse of PP waste are preferable both for diminishing the amount of plastic waste and having significant economic effects. At industrial scale, the separation technique of plastic waste components is based on the float–sink method [17]. Due to the similar density between PP and PE waste, PP is often contaminated with high density polyethylene (HDPE) coming from extrusion (packaging items) [18] and injection (bottle caps) [19] processing technologies. Due to the immiscibility between PP and PE matrices, the mechanical properties of the recycled PP are not enhanced and the use of compatibilizers is required [19]. 

During the mechanical recycling processing, the mechanical properties of PP waste are depreciated compared with those of a virgin polymer. Usually, 5% virgin polymer is used during the processing cycle, as the mechanical properties of PP waste should not deteriorate. Literature data have documented several ways to improve the mechanical properties of PP waste, by irradiation [20], reinforcement with natural fibers [12,21,22,23], agricultural waste [24], montmorillonite [25], sepiolite and zeolite inorganic fillers [26], introduction of long chain branched PP [27], elastomers [28,29,30,31,32,33,34], or non-metallic parts of printed circuit boards [35]. The irradiation technique could be a method for improving the tensile and flexural properties of recycled PP/microcrystalline cellulose (MCC) composites [20]. When a molten resin reservoir was added to a twin-screw extruder, the elongation of break and toughness both increased by 77% as compared with the original PP waste [36]. Another paper reported that the flexural properties, impact strength, and heat deflection temperature (HDT) of kenaf fiber/PP composites with waste expanded PP (EPP) were markedly higher than those with virgin PP. Flexural modulus, flexural strength, impact strength, and HDT properties were enhanced in the range of 20–98%, 25–55%, 26–31%, and 7–12%, respectively, depending on the kenaf fiber content [22]. Belviso et al. [26] observed that recycled PP waste with a natural clay mineral and zeolite showed a decrease in the elongation of composites, due to the filler’s agglomeration leading to the weakening of interfacial interactions. Other researchers reported that the addition of n-clays improved the properties of recycled PP composites used for the treatment of textile dye waste water [37] or in food packaging [38]. 

The interfacial interactions between a polymer matrix and the dispersed fillers are crucial factors in attaining the material’s unique properties. They depend on particle dispersion, surface modification, polymer features, and particle characteristics [39]. Literature data revealed the use of compatibilizers such as maleic anhydride grafted PP (PP-g-MA) to enhance the compatibility between the fiber and the polymeric matrix [12]. Thus, the tensile strength increased from 21.8 MPa (neat recycled PP) to 28.9 MPa in the case of recycled PP/hemp fibers compatibilized with PP-g-MA, while the impact strength recorded an improvement of 9.1% [12]. Moreover, the mechanical properties of recycled PP based on wood and a coupling agent were found to be superior to those of virgin PP and wood [40]. Another study demonstrated that the incorporation of organophilic layered silicates and 20 wt% of PP-g-MA compatibilizer improved the impact and tensile strength of recycled PP reinforced with organophilic layered silicates by 36% and 40%, respectively [41]. 

Thermoplastic elastomers (TPEs) generally possess elastomeric characteristics and can be processed as thermoplastics. Furthermore, TPEs have received much attention, especially for their nano-domain morphology. PP/elastomer composites were successfully used in cable insulation [42,43] and engineering applications, where high impact strength properties are required. The biphasic morphology of PP and elastomer determines the polyolefin modification, which is otherwise difficult to achieve due to incompatible or low-compatibility polymers [44]. In addition, TPEs are expensive. Ghioca et al. [29,30,33] reported that thermoplastic elastomers that provided a viscosity in the melt closer to that of the recovered PP led to high physical-mechanical properties of PP composites. Given that the melt viscosity of the polyisoprene/polybutadiene (PI/PB) phase is higher than that of the PS phase, the reduction or increase in the molecular weight of the (PI/PB) blocks leads to a more pronounced decrease or increase in the melt viscosity of the styrene-isoprene-styrene block copolymers. Thus, optimal properties of the composites will be achieved when the rheological behavior in the melt of the components is as close as possible.

The addition of clay (silicate) to styrene-ethylene-butylene-styrene (SEBS) block copolymer/clay nanocomposites improved the thermal stability of TPE [45]. The loading of clay content up to 20 wt% into recycled PE enhanced the mechanical and thermal properties of a composite material [46]. In a previous paper, we reported the dual effect of glass bubbles and styrene-diene block-copolymers for enhancing the flexibility, Izod impact, processability, thermal stability, weight reduction, storage modulus, loss modulus, and stiffness of recycled PP contaminated with PE [31]. To the best of our knowledge, literature data have not reported studies containing n-clay and styrene-diene block-copolymers co-fillers to reinforce PPW composites. 

The objectives of this paper were to valorize a mixture of PP waste contaminated with HDPE during the recycling process in innovative materials, by blending PPW with block-copolymers and n-clay during the melt processing, and to investigate the mechanical and thermal properties by changing the biphasic, crystalline, and amorphous morphology of the PPW. The aim of adding n-clay and styrene-diene block-copolymers to PPW is to improve the mechanical properties, such as the elasticity and impact strength, as well as the thermal stability. The PPW/elastomer/n-clay composites were characterized by optical microscopy, FT-IR, mechanical measurements (density, tensile, hardness, impact properties, DMA), thermal analyses (DSC, XRD, TG, heat deflection temperature (HDT), VICAT softening temperature (VST)), and compared with neat PPW and PPW/n-clay composites.

## 2. Materials and Methods

### 2.1. Materials 

PP post-production waste contaminated with HDPE (PPW) as pellets was provided by a local Romanian recycler involved in the selective collection, washing, grinding, and granulation steps of polyolefin waste. This matrix was characterized by a density of 0.9948 g cm^−3^, melt flow index (190 °C, 5 kg) of 8.74 g/10 min, tensile strength of break of 24.12 MPa, elongation at break of 28.3%, VICAT softening temperature of 138 °C, HDT of 68 °C, Shore hardness of 63.5 °Sh D, and notched IZOD impact strength at 23 °C of 1.35 kJ m^−2^. Bentonite was purchased from a quarry (Valea Chioarului Mine), Valea Chioarului, Maramureș, Romania, being coded as n-clay in the manuscript. Before processing, the n-clay filler was dried at 60 °C for 4 h. 1,3-Butadiene >99%, styrene >99.9%, and 6-di-tert-butyl-4-methylphenol (TOPANOL OC) were used from Fluka-Sigma-Aldrich (Saint Louis, MO, USA); isoprene 98%, stabilized was acquired from Acros Organics (Geel, Belgium); cycloxexane, pure p.a. was provided by Poch. SA (Gliwice, Poland); and n-butyllithium, solution 1.6 M in hexanes was from Alfa Aestar Gmbh (Karlsruhe, Germany).

### 2.2. Synthesis and Characterization of SBSBC and SISBC

The styrene-butadiene and styrene-isoprene block-copolymers (SBSBC and SISBC) were synthesized using anionic sequential polymerization of monomers in the presence of cyclohexane solution (1.5 moles), and n-butyl lithium (BuLi) as initiator [47]. The advantage of anionic polymerization is obtaining the desired molecular weight of styrene-diene block-copolymers, with well-defined linear and branched structures. PS domains dispersed in the continuous PB or PI elastomer phase ensure crosslinking of the block copolymers. 

**Figure 1:**
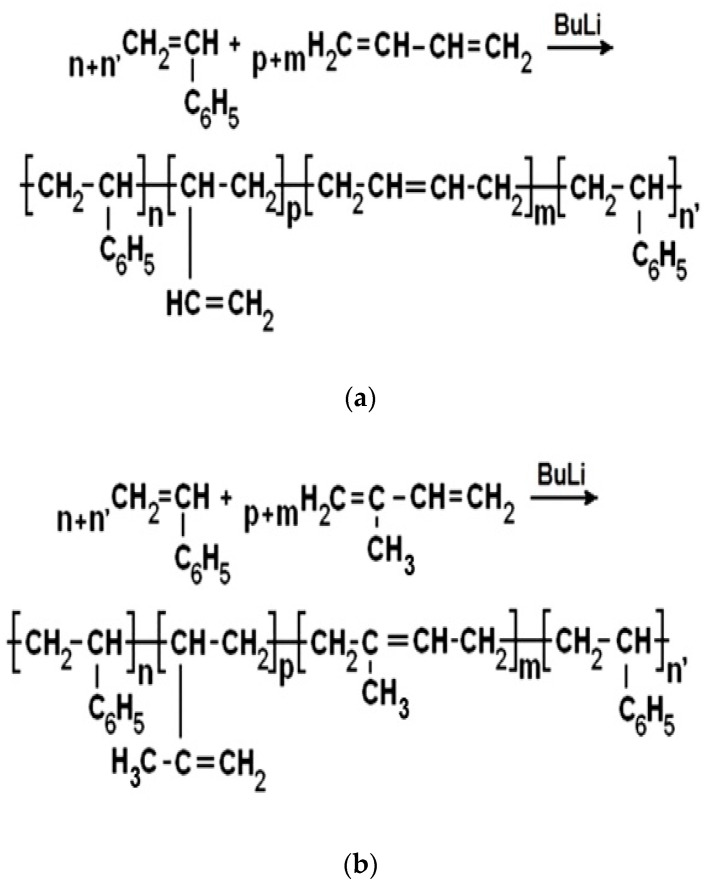
Synthesis of SBSBC (**a**) and SISBC (**b**) by three sequential anionic polymerizations.

First, PS block synthesis was performed and the polystyryl-lithium chains were obtained. Then, the synthesis of the PB/PI blocks was realized by adding a calculated amount of butadiene/isoprene monomer to polystyryl-lithium. Next, the active di-block-copolymer of polystyrene-polybutadienyl-lithium/polystyrene-polyisoprenyl-lithium was reacted with the same amount of styrene used in the synthesis of the first PS block, resulting in SBSBC/SISBC (Figure 1).

Evaluation of the molecular weight, and mechanical and thermal properties, for the synthesized SBSBC and SISBC was performed on 1-mm thick films obtained by centrifugal casting of the elastomer solutions into toluene, using gel permeation chromatography (GPC) and tensile, hardness, melt flow index, and dynamic mechanical measurements (Table 1).

### 2.3. Processing of PPW/SBSBC/n-Clay and PPW/SISBC/n-Clay Composites

Polymer composites were obtained by melt processing in a Brabender Plastograph, at 180 °C and with a rotor speed of 50 rpm of PPW, 10 wt% styrene-diene (SBSBC or SISBC) block-copolymers, and 0, 5, and 10 wt% n-clay, respectively. Degradation of the styrene-isoprene-styrene block copolymer during the processing was avoided by introduction of 2,6-di-tert-butyl-4-methylphenol (TOPANOL OC) in a proportion of 1% in relation to the total amount of mixture. 

Plates of 1-mm and 4-mm thickness, for determining the mechanical and thermal characteristics, were obtained by pressing at a temperature in the range of 185–190 °C for 15 min, under a pressure of 200 bar, followed by sudden cooling of the mold under pressure. In order to eliminate air bubbles during pressing, 2–3 short depressurizations were performed. A neat PPW sample was prepared in the same conditions as the PPW composites and further used as a reference.

### 2.4. Investigation Methods

#### 2.4.1. X-ray Fluorescence and Particle Size Distribution

X-ray fluorescence (XRF) measurement was performed, in order to characterize the nanoclay, using an EDXRF PW4025 energy-dispersive spectrometer, with a detector type MiniPal 2 (PANalytical, B.V., Almelo, The Netherlands). The measurement was performed with a Si-PIN technology, at 20 kV and automatic current intensity, for 300 s, in helium atmosphere. Aluminum and molybdenum filters were used for the analysis of n-clay filler, in order to remove the Rh lines (from the X-ray tube) and other light elements.

Wavelength dispersive X-ray fluorescence spectrometry (WDXRF) was performed for PPW, in order to determine the qualitative and quantitative elementary composition (elements ranging from 17 Cl to 92 U). The detection limit was between 1 ppm and 10 ppb and the accuracy was lower than 0.1–0.5%.

The size measurement of n-clay particles was performed using a dynamic light scattering (DLS) technique by means of a Zetasizer Nano ZSP (Malvern Instruments, Worcestershire, UK). The scattered light was collected at 173°, with a red laser wavelength of 632.8 nm (He/Ne). Then, 0.1 g of n-clay powder was immersed in 5 mL of ultra-pure water and sonicated for 5 min. Then, 3 suspension drops were dispersed in 10 mL solution 1 mM NaCl, homogenized again, and analyzed using a 12-mm cell (DTS 0012). Measurements of Z-average, size, and polydispersity index were performed in triplicate, and the results are expressed as mean values ± standard deviation.

#### 2.4.2. Microscopic Observation 

A Leica DM 2500 M microscope (Leica Camera AG, Wetzlar, Germany) equipped with a digital camera at 500× magnification was used to observe the morphology of the PPW composites.

#### 2.4.3. ATR-FT-IR Analysis

The spectral characteristics of the PPW composites were recorded using an INTERSPEC 200-X Fourier transform infrared spectrophotometer (Interspectrum, Tartumaa, Estonia) with an ATR device, by scanning the wavenumber range from 3500 to 750 cm^−1^, at an angle of incidence of 45° and a resolution of 4 cm^−1^.

#### 2.4.4. Differential Scanning Calorimetry (DSC)

Thermal analysis of PPW composites was carried out using a DSC 823^e^ calorimeter supplied by Mettler Toledo (Greifensee, Switzerland), previously calibrated with an indium standard. Samples weighing between 8 and 10 mg were packed in aluminum pans and placed in the DSC cell. The samples were first heated from ambient temperature to 195 °C at a rate of 10 °C min^−1^, kept 2 min at 195 °C, in order to erase any previous thermal history, and then cooled to ambient temperature at the same heating rate. The crystallization temperature (T_c_), melting enthalpy (ΔH_m_), and peak melting temperature (T_m_) were evaluated from the cooling and the first heating cycle, respectively. The degree of crystallinity (*X_c_*) was calculated from DSC curves, according to Equation (1)

(1) $$X_{c}=\frac{\Delta H_{m}}{\Delta H_{m\;}^{0}\times \left(1-w\right)}\times 100\%$$

where ΔH_m_ is the heat of fusion for blend (J/g); $\Delta H_{m}^{0}$ is the theoretical heat of fusion for 100% crystalline PP and HDPE, respectively, which recorded a value of 138 J g^−1^ [12] and 290 J g^−1^ [48], and w is the fraction of mineral filler from sample [49].

#### 2.4.5. X-ray Diffraction (XRD)

X-ray analysis of the recycled PPW was carried out with an Panalytical X’Pert PRO MPD diffractometer (Almelo, The Netherlands). The wavelength (λ) for the CuKα-Ni filtered irradiation was 1.54065 nm. Diffraction angle 2θ was scanned from 10° to 90°, at a scanning rate of 5 °C min^−1^, using a Pixcel detector with 256 canals. The diameter of the crystallite of rPP composites was determined using the Debye-Scherrer equation [50].

(2) $$D=\frac{K\times \lambda_{\mathrm{Cu}-K\alpha }}{\cos\theta \times \mathrm{FWHM}}$$

where D is the average crystallite size, nm; K is the crystallite shape factor (0.89); λ_Cu−Kα_ is the X-ray wavelength; FWHM is a peak width at half-maximum intensity, 2θ; and θ is the scattering angle.

#### 2.4.6. TGA

TGA of samples was performed using a TGA Q5000IR equipment (TA Instruments, New Castle, DE, USA), in accordance with Hi-Res MTGA method-sensitivity 1, modulated ± 3 °C for 120 s, with a speed of 10 °C min^−1^. The average weight of the samples in the range of 6–10 mg was placed into platinum-HT 100 µL sample pan and exposed to three heating cycles from 25 °C to 310 °C, 310–560 °C, and 570–700 °C, respectively, to obtain the maxim temperature (T_max_) and weight reduction under nitrogen using a flow rate of about 50 mL min^−1^. The residue % at 700 °C, the maxim temperature (T_max_) and mass loss associated, as well as the onset temperature at decomposition and mass loss, were also measured. 

#### 2.4.7. Mechanical Properties

Tensile tests (recording tensile strength and elongation at break) were carried out using a Universal Traction Machine (Instron 8802, Norwood, MA, USA). Five dog-bone shaped specimens for each sample were measured at a crosshead rate of 50 mm min^−1^ (ISO 527-1:2019).

Hardness measurements were performed on a Shore D durometer (Zwick Roell, Ulm, Germany), in accordance with ISO 868:2003. Test specimens of 4 mm thickness and a loading force of 4.536 kg were used. The average value of five measurements was taken into consideration for each sample.

The VICAT softening temperature (VST) and heat deflection temperature (HDT) measurements were conducted using an HDT/VICAT Softening Point Apparatus (CEAST Test Equipment, Pianezza, Italy) equipped with a silicone oil bath. VST determination was performed according to ISO 306:2004 (Method A50), at a heating rate of 50 ± 5 °C h^−1^, using three test specimens for each sample with a thickness of 4 mm. HDT measurement was conducted according to ISO 75:2020, at a heating rate of 120 ± 10 °C h^−1^ and a load of 1.8 MPa using two specimens with (80 × 10 × 4) mm dimensions for each sample. 

The V-notched Izod impact test was realized at room temperature, using an Izod impact tester (Ceast, Pianezza, Italy), with a hammer of 2 J, which was operated according to ISO 180:2019. Five rectangular test specimens with a length of 80 mm, width of 10 mm, and height of 4 mm were employed for each sample, to obtain the average impact strength Izod impact (a_IN_), according to Equation (3).

(3) $$a_{\mathrm{IN}}=\frac{E_{c}}{h\times b}\times 10^{3}\;\mathrm{kJ}/m^{2}$$

where E_C_ is energy used for breaking of specimen, J; h is thickness of specimen, mm; and b is width of specimen under notch, mm.

#### 2.4.8. Dynamic Mechanical Analysis (DMA)

The viscoelastic properties, such as storage modulus (E′), loss modulus (E″), loss factor (tan δ), and stiffness of PPW composites as a function of temperature were determined using a DMA Q800 equipment (TA Instruments Inc., New Castle, DE, USA), which operated with dual cantilever bending. Data processing was performed with Universal Analysis 2000 software (Version 4.5 Build 4.5.0.5, New Castle, DE, USA). Rectangular specimens having a width of 10.56 mm, a thickness of 4 mm, and a length of 60 mm were examined according to the testing program conducted, from ambient temperature to 150 °C, with a constant heating scan of 3 °C min^−1^, a frequency of 1 Hz, and amplitude of 15 µm. 

## 3. Results

### 3.1. Characterization of n-Clay and PPW

**Figure 2:**
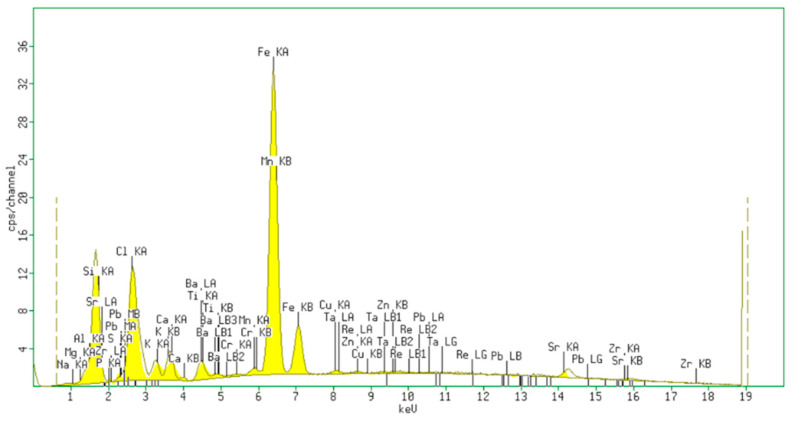
Energy-dispersive X-ray fluorescence spectrum of the n-clay.

The chemical composition and oxide level of commercial n-clay were evaluated by X-ray Fluorescence (XRF) Spectroscopy (Figure 2), as is depicted in Table 2.

The presence of Si, Na, Al, Mg, and Fe cations in n-clay composition could play a surfactant role, allowing a decrease of the surface tension at the interface between the PP chain and elastomer.

**Figure 3:**
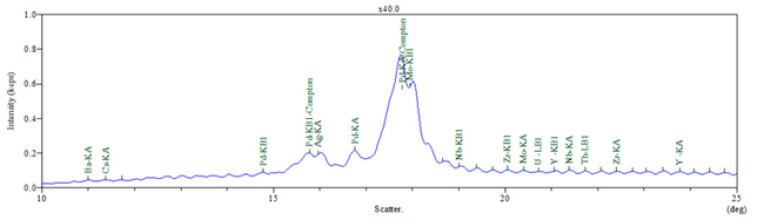
WDXRF for PPW.

The elemental composition of PPW resulting from the WDXRF analysis is illustrated in Figure 3 and Table 3.

The elemental analysis recorded for PPW, according to Figure 3 and Table 3, shows the presence of different percentages of metals, halogens, and oxides in the initial plastic material. As expected, PPW contained elements such as Al, Mg, Ca, Si, and P, which were attributed to the addition of aluminum/magnesium silicates or hydroxides, and calcium carbonate or talc, respectively, as inorganic fillers into the plastic matrix processing. Elements such as Ti, K, or Fe are usually used in polyolefin processing as thermal aging stabilizers, coloring agents, and fillers. Halogens such as Br, Cl, and I come from the flame retardants used [35]. Chemical elements such as Ti, Si, Br, Al, S, Cl, Fe have also been detected in HDPE product waste [51], suggesting their presence in PPW compositions. 

The presence of the highlighted compounds could modify the morphology of the PPW composites acting as heterogeneous nucleating agents, to improve the nano-clay dispersion.

**Figure 4:**
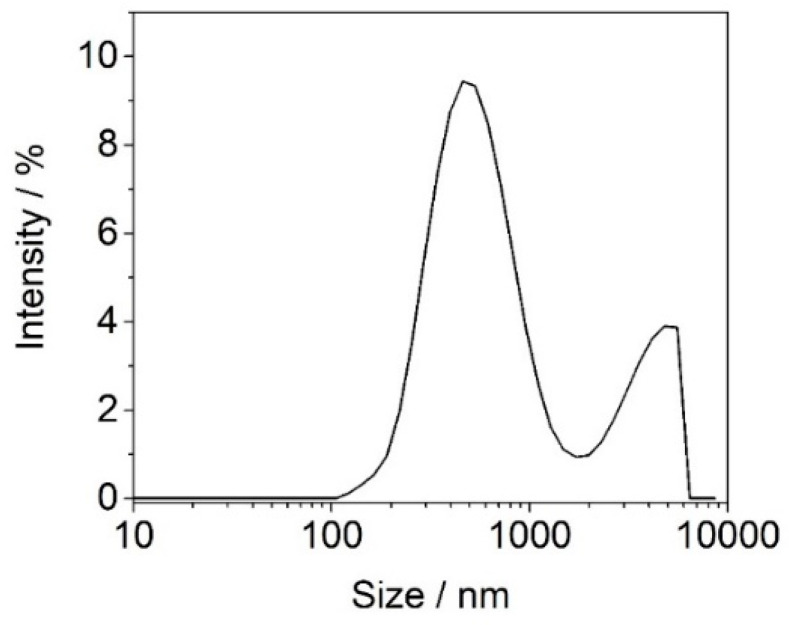
Size distribution of clay particles.

Figure 4 illustrates the dimensional distribution and size of the clay particles determined using the laser diffusion technique (DLS).

The n-clay had a dispersity index (Pdi) of 0.412 and two peaks with different intensities, one of 572.9 ± 295.9 nm (>78% distribution) and another of 3885 ± 1170 nm (21.7% distribution). The high specific surface area of n-clay (Z-Average 603.8 nm) was associated with high specific surface properties, leading to improved properties as compared with micrometer-sized particles [39]. 

### 3.2. Optical Microscopy

**Figure 5:**
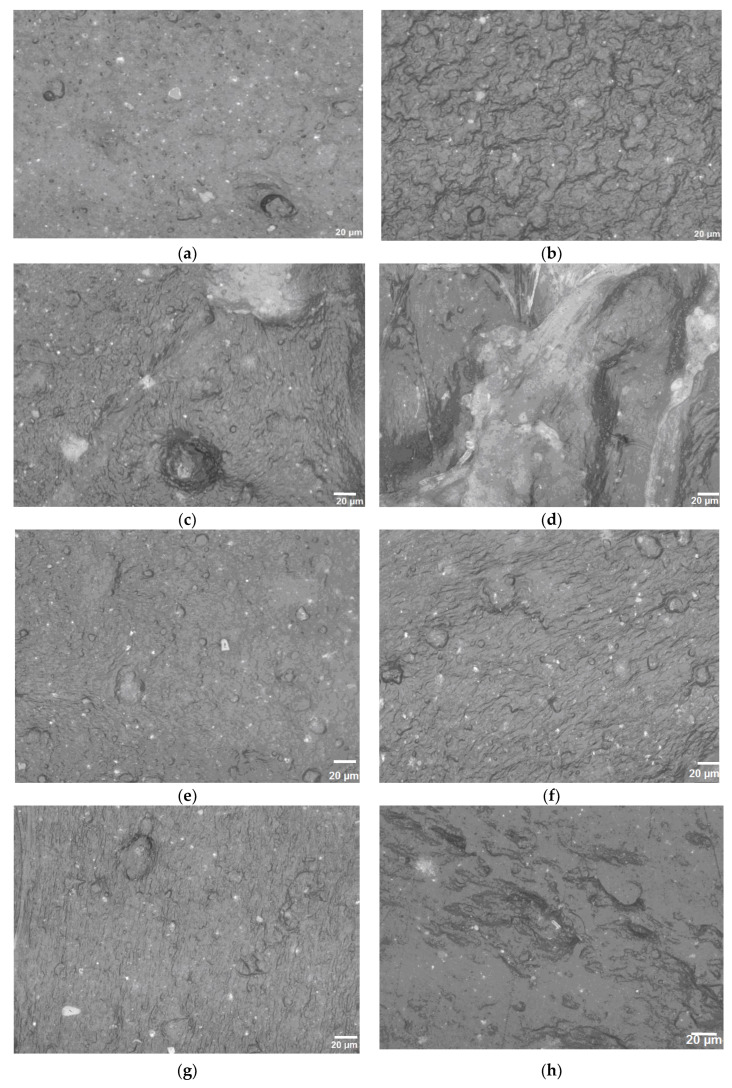

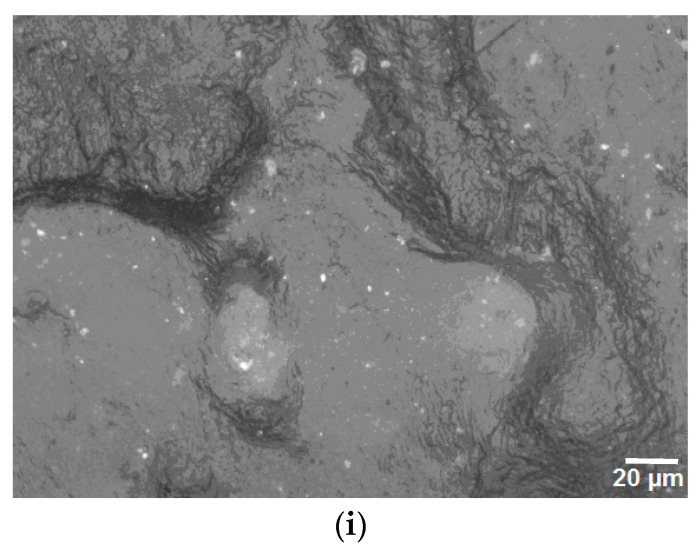
Optical images (500×) for: PPW (**a**); PPW/SBSBC (**b**); PPW/n-Clay 5% (**c**); PPW/n-Clay 10% (**d**); PPW/SBSBC/n-Clay 5% (**e**); PPW/SBSBC/n-Clay 10% (**f**); PPW/SISBC (**g**); PPW/SISBC/n-Clay 5% (**h**); PPW/SISBC/n-Clay 10% (**i**).

Micrographs of the PPW composites, obtained using optical microscopy analysis are shown in Figure 5.

The absence of the elastomer promoted gap formation between the hydrophobic PP matrix and the hydrophilic n-clay (Figure 5c,d). This behavior could be also due to the poor mixing that occurred during the melt processing between the PPW and n-clay with an impact on the decrease of the mechanical properties. Meanwhile, the addition of n-clay tended to form aggregates in the absence of elastomer, due to the hydroxyl groups on the surface of n-clays, which formed hydrogen bonds and attracted each other. The SBSBC or SISBC appeared as dispersed droplets in the PPW matrix (Figure 5b,g), encapsulating the n-clay particles (Figure 5e,f,h,i). This composite morphology enhanced the efficiency of the n-clay–thermoplastic matrix blending compared to the composites without elastomers and would improve the impact strength property of PPW composites. SBSBC exhibited a more effective adhesion between the polymeric matrix and n-clay, as compared with SISBC. In addition, the surface energy, wettability, and interfacial adhesion between phases led to the PPW/elastomer/filler composite’s phase structure [34]. 

In the case of complex blends, their morphology and phase behavior are influenced and determined by various factors, such as: the distribution of the dispersed phase in the matrix, reciprocal solubility, the nature of the intermolecular interactions, and the potential reactions between the components that appear during both processing and heating at various temperatures. These factors also influence the ratio between the crystalline and amorphous fractions in semicrystalline polymers, the variation of glass transition temperatures, the melting behavior, thermo-oxidative behavior, etc. [44].

### 3.3. ATR-FT-IR Analysis

**Figure 6:**
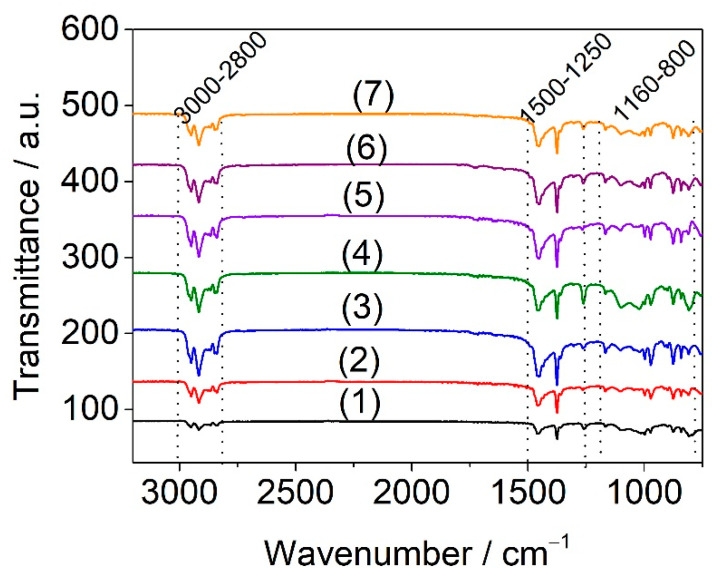
ATR-FT-IR spectra of PPW (1), PPW/SBSBC (2), PPW/SBSBC/n-Clay 5% (3), PPW/SBSBC/n-Clay 10% (4), PWP/SISBC (5), PPW/SISBC/n-Clay 5% (6), and PPW/SISBC/n-Clay 10% (7) samples.

The infrared spectra for PPW, PPW/elastomer, and PPW/elastomer/n-clay samples recorded in ATR mode are depicted in Figure 6.

In spectra shown in Figure 6 can be observed the three main spectra regions: 3000–2800 cm^−1^, 1500–1250 cm^−1^, and 1160–800 cm^−1^. The PPW sample displayed characteristic peaks at 997 cm^−1^ and 975 cm^−1^ attributed to the crystalline and amorphous structure of PP [36], 2830 cm^−1^ related to the symmetrical stretching of CH_2_ (amorphous and crystalline phases), and 2900 cm^−1^ and 2950 cm^−1^ assigned to the asymmetric stretching bands of CH_2_ (amorphous phase) [52]. The absorption bands at 1461 cm^−1^ and 1377 cm^−1^ are the -CH_3_- (symmetric bending), while low intensities of absorption bands are observed at 1159 cm^−1^ (CH bending, CH_3_ rocking, C-C stretching) [53]. The bands at 800 cm^−1^, 871 cm^−1^ and 840 cm^−1^ correspond to the vibrations of terminal unsaturated CH_2_ groups, CH_2_ rock, and C-CH_3_ stretching, specific to isotactic PP [54]. 

The specific bands assigned to the polybutadiene, polyisoprene, and polystyrene components from SBSBC and SISBC structure are evidenced by the increase in intensities at 1020 cm^−1^ and 1261 cm^−1^, and width of the band at 1461 cm^−1^ [32]. The characteristic absorption of Si-O at around 1000 cm^−1^ was not seen, denoting the missing of interactions between components. Another issue observed from Figure 6, is that the composites were not degraded during processing, as proven by the absence of the specific band at 1720 cm^−1^ associated with the carbonyl stretching (C=O).

### 3.4. Differential Scanning Calorimetry (DSC)

**Figure 7:**
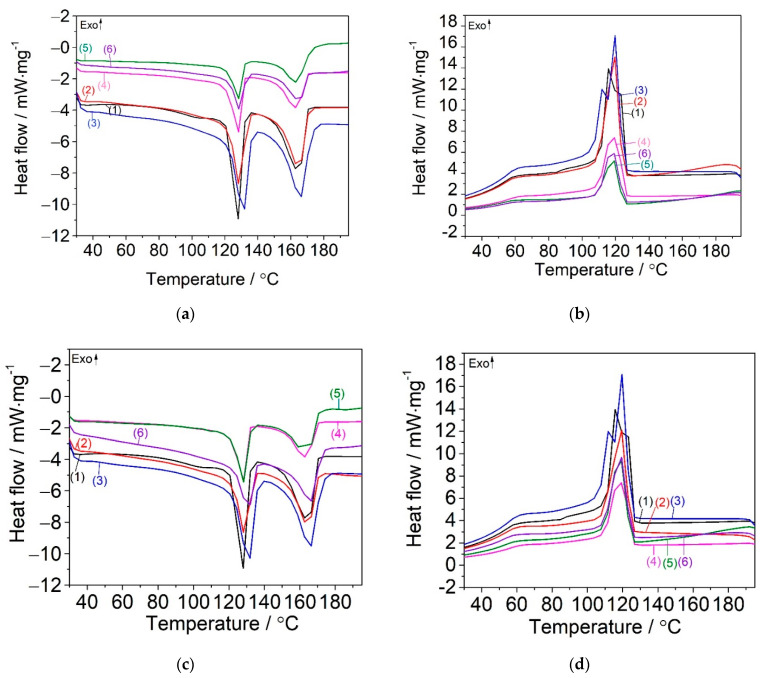
DSC curves of PPW (1), PPW/SBSBC (2), PPW/n-Clay 5% (3), PPW/n-Clay 10% (4), PPW/SBSBC/n-Clay 5% (5), PPW/SBSBC/n-Clay 10% (6) samples (**a**) melting and (**b**) crystallization steps; PPW (1), PWP/SISBC (2), PWP/n-Clay 5% (3), PPW/n-Clay 10% (4), PPW/SISBC/n-Clay 5% (5), PPW/SISBC/n-Clay 10% (6) samples (**c**) melting and (**d**) re-crystallization steps.

The DSC curves of all analyzed samples are given in Figure 7. Table 4 gathers the DSC parameters obtained from the evaluation of DSC curves. The indexes 1 and 2 from Table 4 refer to PE and PP, respectively.

The unfilled PPW showed a T_m_ of 164.6 °C, similar to the value reported in the literature for other PP wastes [12]. Two melting endothermic peaks (T_m_) were observed for the PPW composites, one related to the melting of high density polyethylene (HDPE) at T_m1_ ~129 °C [38,55], and another one to the melting of PP at T_m2_ ~164 °C (Figure 7a). Other authors also reported the additional melting peak ascribed to HDPE during the heating process [27,56,57,58]. From the heat fusion of the PE (ΔH_m1_), the degree of crystallinity of PE in the total PPW can be estimated. Based on the hypothesis that the crystalline phase from PE is the same as the amorphous ones [19,59], ~20% HDPE was found to be in the recycled PP. Taking into consideration that a part of the crystalline PPW has been replaced by SBSBC, n-clay or both, the T_m_ values (especially T_m1_) for PPW/SBSBC/n-clay composites evidenced an increase in relation to neat PPW, due to the good dispersion of n-clay filler encapsulated into the butadiene blocks. Both PPW/SISBC and PPW/SBSBC composites showed a degree of crystallinity of ~20.6% and 19.5%, respectively (Table 3), meaning that the presence of isoprene and butadiene blocks in the elastomers structure created a worse interfacial compatibility with the PPW polymeric matrix, leading to a reduction of the active centers of crystallization.

The introduction of 5 wt% and 10 wt% n-clay to PPW decreased the degree of crystallinity of PP from 24.1%, in the case of neat PPW, to 22.0% and 20.4%, respectively, (Table 4) due to the chain mobility reduction [60]. The same decrease of the crystallinity was reported in the case of the introduction 2% clay into HDPE/pine flour composite [60] and 5% clay into the PP matrix [61]. The reduction in the degree of crystallinity was explained by the adding of clay, which reduced the PP macromolecular chain mobility and, thus, avoided an arranged alignment of the crystal lattice [60,61]. 

The encapsulated structure morphology of PPW composites was also proven by the examination of T_c_ and T_m_ parameters obtained during the first heating and cooling scans. Figure 7b,d shows the cooling cycles for PPW/SBSBC/n-Clay and PPW/SISBC/n-Clay composites, from which the two transitions corresponding to PP and PE crystallization temperatures were recorded. The T_c2_ gently decreased with the incorporation of n-clay and elastomer, which signified a slow interaction of the co-fillers with the PP matrix. The addition of both co-fillers to PPW had an increasing effect on the melting temperatures, which were observed to be more accentuated for SISBC/n-Clay 10% and SBSBC/n-Clay 10% systems. The encapsulation of 5% n-clay and 10% n-clay, respectively, into SISBC or SBSBC created a greater disruption of the microcrystalline PP network (the high degree of crystallinity of PP recorded values of 20.8% and 27.5%), maybe due to the increased entanglements of SISBC compared with SBSBC and hard dispersion of n-clay. The decrease of both T_c_ and Xc parameters was reported in the case of adding tobacco stalk flour (TSF) and magnesium oxysulfate whiskers (MOSw) to recycled PP, making the interface compatibility of the composite worse [24]. 

A nucleating effect of n-clay was only observed for the PPW/SBSBC/n-Clay 5% composite (the degree of crystallinity for PP increased to 27.5%). A strong nucleation effect of filler was reported in the case of PP/ethylene–octene copolymer (EOR)/CaCO_3_ composites, which exhibited a separate dispersion morphology [34].

### 3.5. X-ray Diffraction

**Figure 8:**
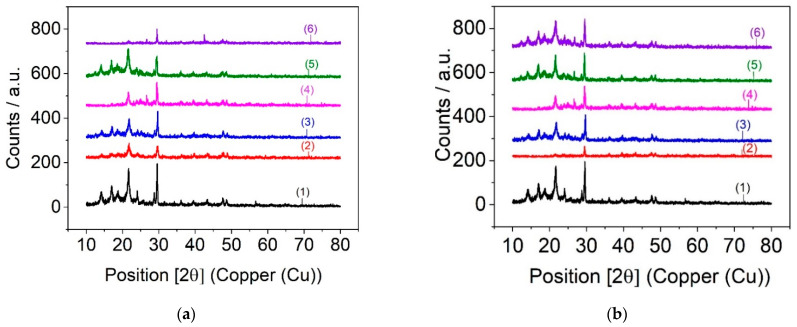
XRD patterns of PPW (1), PPW/SBSBC (2), PPW/n-Clay 5% (3), PPW/n-Clay 10% (4), PPW/SBSBC/n-Clay 5% (5), and PPW/SBSBC/n-Clay 10% (6) samples (**a**) and PPW (1), PPW/SISBC (2), PWP/n-Clay 5% (3), PPW/n-Clay 10% (4), PPW/SISBC/n-Clay 5% (5), PPW/SISBC/n-Clay 10% (6) samples (**b**).

XRD diffraction patterns for PPW, PPW containing 5% and 10% n-clay, PPW containing SBSBC and SISBC, and PPW including n-clay and elastomer were registered, in order to measure the average crystallite size (Figure 8 and Table 5).

The X-ray profile in Figure 8 indicates the approximately equal intensities of signals characteristic for crystalline polymers, together with the amorphous character observed for the synthesized block copolymer. The peaks of 2-theta at 16.9°, 18.4°, 25.3°, and 28.6° detected in pure PPW were assigned to the crystalline phase of PP [35], while the peaks appearing at 21.5° and 23.2° were due to the PE [36,60]. 

From Table 5, it can be seen that the crystallite size depended on the type of elastomer and the amount of n-clay. Crystallite size determinations using X-ray diffraction for the PPW composites confirmed the modification of the microcrystalline network of PPW. The average size of the crystallites decreased progressively with the introduction of the block copolymer into the polyolefinic matrix. It was observed that the addition of 5% n-clay and elastomer decreased the crystallite size to 28.98 nm in the case of SBSBC and 16.12 nm in the case of SISBC. This decrease can be explained by the increase in the active centers of crystallization, which had the consequence of decreasing the crystallite size. Second, the introduction of elastomer and n-clay into the system diluted the polyolefin matrix, thus, reducing the degree of alignment of the polyolefinic macromolecules, which led to an overall reduction of the crystalline phase of the composites. An increase in crystallite size was observed with introduction of 10% n-clay and elastomer into the PPW. The increase in crystallite size for PPW/SBSBC/n-Clay composites compared with those of PPW/SISBC/n-Clay composites can be explained by the good dispersion of particles of SBSBC into the polyolefin matrix.

### 3.6. Thermogravimetric Analysis (TGA)

**Figure 9:**
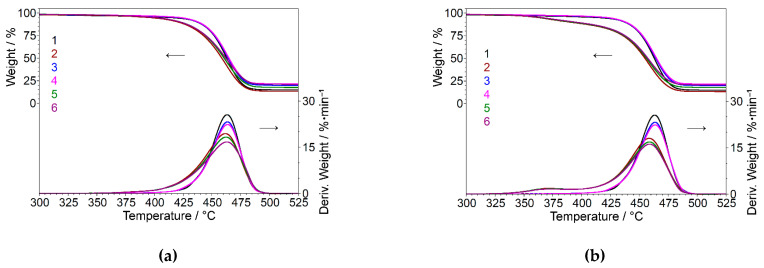
TGA analysis for PPW/SBSBC/n-Clay composites (PPW (1), PPW/SBSBC (2), PPW/n-Clay 5% (3), PPW/n-Clay 10% (4), PPW/SBSBC/n-Clay 5% (5), PPW/SBSBC/n-Clay 10% (6)) (**a**) and PPW/SISBC/n-Clay composites (PPW (1), PWP/SISBC (2), PWP/n-Clay 5% (3), PPW/n-Clay 10% (4), PPW/SISBC/n-Clay 5% (5), PPW/SISBC/n-Clay 10% (6)) (**b**).

To evaluate the thermal stability of the PPW composites, TGA was employed from 25 °C to 700 °C, at a constant heating rate. Figure 9 shows the TGA and DTG curves for PPW/SBSBC/n-Clay and PPW/SISBC/n-Clay composites.

Figure 9 shows the thermal stability of PPW/SBSBC and PPWSISBC loaded with two contents of n-clay, as estimated by thermogravimetric analysis. The initial degradation temperature showed in Table 6 reveals that the incorporation of elastomer and n-clay into PPW matrix did not lead to an improvement of the thermal stability of the composites, as compared with the thermal stability of pure PPW. The same results were obtained by adding unoriented talc to a commercial isotactic PP [62]. However, the processing temperature for PPW composites is below the temperature of the onset of degradation of each component.

N-clay started to degrade above 200 °C [25], and continued its degradation with a lower weight loss till 560 °C, a total mass loss of 90.26% being recorded at 700 °C. Delva et al. [25] showed that the degradation of organoclay began at 205 °C and finished at 400 °C.

The PPW showed a high weight loss (83.42%) in the temperature range of 310–560 °C and 9.59% residue at 700 °C, due to the presence of inorganic fillers, such as talc and CaCO_3_, added during the initial melt process [31].

The low mass loss of the PPW composites of ~1.2% recorded at a temperature of 310 °C proved the excellent thermal stability of the samples with the reinforcing step. The measurements also indicated that the thermal stability of PPW/SBSBC/n-clay is higher than that of PPW/SISBC/n-clay, due to the morphology of SISBC, which showed a maximum weight loss (98.61%) at 375.2 °C. The residual ash content at 700 °C for composites containing PPW, elastomer, and n-clay was in the range of 8 to 16 wt%, depending on the type of elastomer and content of n-filler in the composites. 

### 3.7. Mechanical Properties

The results obtained from the mechanical characterization of PPW/elastomer, PPW/n-clay, and PPW/elastomer/n-clay are shown in Table 7 (tensile strength and elongation at break, hardness, VST, HDT, and Izod impact strength).

The styrene-diene block-copolymers introduced into the PPW matrix by melt blending not only modified the crystalline phase (Table 4), but also the amorphous phase of the polyolefin. In the amorphous phase, the block-copolymers dispersed in the form of well-defined domains played the role of a plasticizer, with its elasticizing effect, the specific consequence being an increase of the elongation at break, simultaneously with a reduction in tensile strength (Table 7). The modification of the crystalline network of the PPW (Table 4) produced a decrease of its friability and allowed a more uniform dissipation of the mechanical deformation effect during the traction of the material, thus leading to an increase in the tensile strength of the composites. The dispersed elastomeric domains in the amorphous phase of PPW acted as a plasticizer, leading to a significant increase in the flexibility of the composites, in accordance with similar property findings reported in other papers [63,64]. Compared with neat PPW, the highest enhancements in elongation at break were registered for PPW/SISBC (70.14 ± 3.7%), and PPW/SBSBC (64.32 ± 4.5%), similarly to PPW/SISBC/n-Clay 5% (64.32 ± 4.7%).

The modification effect was influenced by the composition of the styrene-diene copolymers, its amplification being controlled by the rheological behavior in the melt of the elastomers. Thus, the SISBC with the melt viscosity closest to that of PPW gave the most balanced properties of composites, with maximum values of tensile strength and elongation at break, as can be seen from Table 7. It can be observed that the PPW/SBSBC/n-Clay composites recorded increases in impact strength compared with neat PPW of 62 ± 2% and 31.8%, respectively, while the PPW/SISBC/n-Clay composites registered a remarkable impact strength, with an increase of 140% and 59%, depending on the amount of n-clay. This increase was a consequence of the interfacial adhesion between the components of PPW composites, which absorbed and transferred the impact energy [65]. 

Consequently, the greatest modification effect of PPW was produced by SISBC/n-clay, which showed higher values of impact resistance, but also for tensile strength and elongation at break. However, it should be noted that SBSBC also produced a significant improvement in the physical-mechanical properties of the PPW composites, compared to the properties of unreinforced PPW.

The addition of elastomers to the PPW matrix softened the materials at the surface, by changing the crystalline/amorphous ratio, as reflected in the slightly decreased values for hardness (Table 7), but the subsequent incorporation of n-clay led to surface reinforcement, especially for a content of 10%. Regarding the surface mechanical properties, a similar trend as for the hardness property was observed for the VST results. The composites containing both types of elastomers registered the lowest VST values compared with neat PPW, a fact attributed to their improved flexibility, with direct consequence of easier needle penetration at lower temperatures [66]. The evolution of HDT results was similar to that of the VST, being influenced by the stiffness and crystallinity changes of the studied composites, and as a function of the additives used.

### 3.8. Dynamic Mechanical Analysis (DMA)

**Figure 10:**
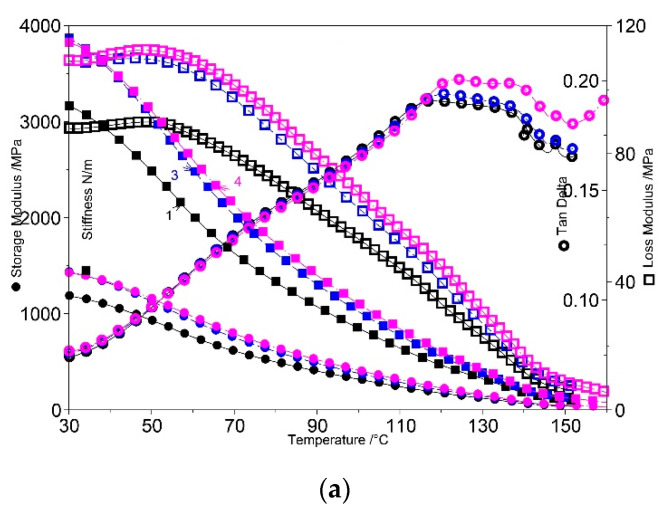

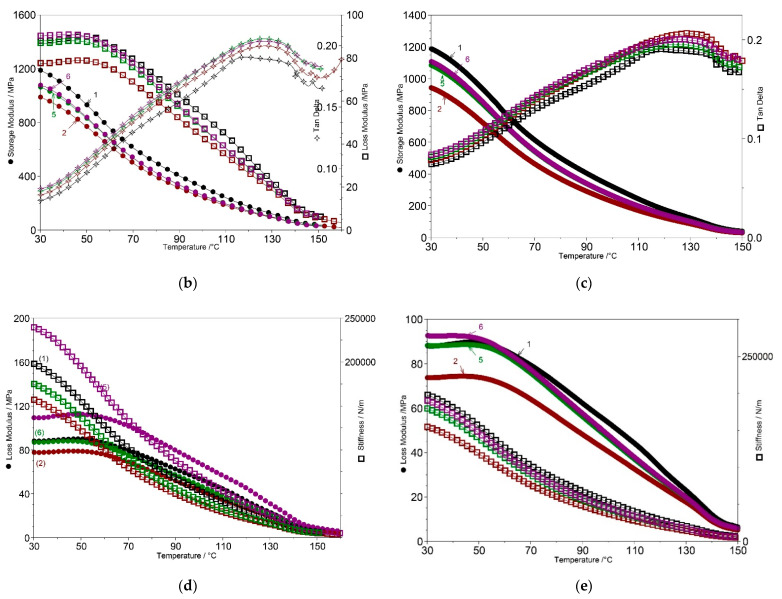
Dynamic mechanical analysis (DMA) analysis. Storage modulus (●), Stiffness modulus (◾), tan δ (🞉), and loss modulus (◽) as a function of temperature (1-PPW (black color), 3-PPW/n-Clay 5% (blue color), 4-PPW/n-Clay 10% (purple color)) (**a**); Storage modulus (●), tan δ (⯎), and loss modulus (◽) as a function of temperature (1-PPW (black color), 2-PPW/SBSBC (wine color), 5-PPW/SBSBC/n-Clay 5% (olive color), 6-PPW/SBSBC/n-Clay 10%) (purple color) (**b**); Storage modulus (●), and tan δ (◽) as a function of temperature (1-PPW (black color), 2-PPW/SISBC (wine color), 5-PPW/SISBC/n-Clay 5% (olive color), 6 PPW/SISBC/n-Clay 10% (purple color)) (**c**); Loss modulus (●) and stiffness (◽) as a function of temperature (1-PPW (black color), 2-PPW/SBSBC (wine color), 5-PPW/SBSBC/n-Clay 5% (olive color), 6 PPW/SBSBC/n-Clay 10% (purple color)) (**d**); Loss modulus (●) and stiffness (◽) as a function of temperature (1-PPW (black color), 2-PPW/SISBC (wine color), 5-PPW/SISBC/n-Clay 5% (olive color), 6-PPW/SISBC/n-Clay 10% (purple color)) (**e**).

DMA analysis is very useful for evaluating the performance of PPW-based samples under stress and temperature. The effect of n-clay and synthesized block-copolymers on the viscous and elastic behaviors of PPW was investigated by DMA in the temperature range of 30–150 °C. Figure 10a–e shows the storage modulus (E′), tan δ (loss factor), loss modulus (E″), and stiffness versus temperature for the obtained composites.

The addition of n-clay in the PPW matrix resulted in a noticeable increase in stiffness, as reflected in the increase in storage modulus values compared to the neat PPW sample, due to the reduced mobility of the polymer chain segments. A high reinforcement in all composites was recorded at 90 °C, when the storage modulus showed an improvement when increasing the amount of n-clay (Figure 10a). Loss modulus curves of the function of temperature recorded a peak, visible around 50 °C, attributed to α relaxation in PP, due to the slip and rotation of the crystalline lamellae [67]. This relaxation was enhanced in the presence of n-clay and more observable for the 10 wt% amount. Similar findings were recorded by Majka et al. [68].

The incorporation of synthesized elastomers in the PPW phase led to a decrease of storage modulus (*E*′), attributed to an increase of elasticity, with a more accentuated effect being registered in the case of SISBC (289 MPa at 90 °C) compared with PPW (402 MPa) (Figure 10b,c). The storage modulus versus temperature curves measured at 90 °C (rubbery region) for PPW/elastomer samples (Figure 10b,c) revealed a lower storage modulus (289.3 MPa) for PPW/SISBC in comparison with the PPW/SBSBC composite (313.4 MPa). This can be explained by the entanglement of polybutadiene chains from SBSBC, which were stronger than those of polyisoprene from SISBC. Stiffness also decreased with the incorporation of elastomer. The molecular chains moved more easily under stress because the elastomers dispersed in PPW could transfer and homogenize the external force and increase the elasticity in the melt state.

The tan δ peaks became broader with increasing n-clay content (Figure 10a). Tan δ values increased with the introduction of n-clay and elastomer into the PPW matrix. This reached the maximum at 126.15 °C (0.2054 MPa) and 125.11 °C (0.20003 MPa) for PPW/SBSBC/n-clay 5% and PPW/SISBC/n-clay 10% composites, respectively. 

The recent COVID-19 pandemic affected humanity by inducing health issues, socio-economic crisis, and environmental concerns. An increased percentage of polyolefins have been used since 2019, especially in specific medical applications or personal protection equipment (PPE) [69]; therefore, their valorization by recycling or reuse is of high value, in view of the safe and economic management of plastic waste. 

The materials developed within this study were designed to consider the current concepts of worldwide political directives regarding environmental protection and the circular economy, which aim to reduce waste to a minimum by recycling existing materials and products as far as possible. 

## 4. Conclusions

Styrene-butadiene block-copolymers (SBSBC) and styrene-isoprene block-copolymers (SISBC) were synthesized via anionic three-stage sequential polymerization initiated with n-butyl lithium, with the purpose of improving the performance of PPW, a mixture of PP with HDPE waste, together with n-clay being used as plasticizers, with melt processing technology. The data revealed that the best mechanical properties were provided by the SISBC/n-clay co-fillers (increased elongation, impact strength, storage modulus, loss modulus, and stiffness). The plasticizer mechanism was influenced by both the biphasic morphology of block-copolymers and n-clay adhesion at the polybutadiene/polyisoprene phase.

The introduction of elastomer and n-clay into the PPW composition led to minor changes in melting temperature (T_m_) compared with neat PPW, as seen from the DSC data. The degree of crystallinity registered a decrease for composites with 5% and 10% n-clay, due to the encapsulating of filler into the elastomer, influencing the mechanical properties.

Our findings revealed that innovative materials with good performance and significant potential can be developed by reuse of municipal PP post-consumer waste containing PE as a pollutant and with incorporation of n-clay, with possible benefits for environmental protection.

Future studies will consist in testing a larger number of thermoplastic elastomers, with different microstructures, and reinforcing agents with various n-clay types.

## Figures and Tables

**Table 1:** Characteristics of the synthesized styrene-diene block-copolymers.

Characteristic	SBSBC	SISBC
PS content (wt%)	30.2	30.5
PS block molecular weight (g/mol)	12,650	18,700
PB block molecular weight (g/mol)	118,500	
PI block molecular weight (g/mol)		85,000
Molecular mass (g/mol)	180,500	122,500
Tensile strength (MPa)	19.80	7.60
Elongation at break (%)	840	1440
Hardness (°Sh A)	42.5	38.5
Melt flow index at 190 °C, 5 kg (g/10 min)	7.4	8.6
Tg for PB phase (°C)	−76	
Tg for PI phase (°C)		−60
Tg of PS phase (°C)	86	90

**Table 2:** Major elements and oxide levels of the n-clay.

Element	%	Oxide	%
Silicon (Si)	44.40	SiO_2_	51.10
Phosphorus (P)	19.60	P_2_O_5_	19.00
Chloride (Cl)	8.43	SO_3_	7.93
Sulphur (S)	8.19	Na_2_O	7.00
Natrium (Na)	7.00	Al_2_O_3_	6.30
Aluminum (Al)	5.50	MgO	4.10
Magnesium (Mg)	3.70	Fe_2_O_3_	0.90
Iron (Fe)	1.85		

**Table 3:** Elemental composition of PPW.

Element	%	Oxide	%
Titanium (Ti)	2.86	TiO_2_	0.17
Calcium (Ca)	2.64	SiO_2_	0.12
Silicon (Si)	1.02	Al_2_O_3_	0.11
Aluminum (Al)	0.84	CaO	0.05
Bromine (Br)	0.63	MgO	0.04
Chloride (Cl)	0.43	P_2_O_5_	0.01
Magnesium (Mg)	0.40	SO_3_	0.01
Potassium (K)	0.25		
Iodine (I)	0.24		
Sulphur (S)	0.22		
Iron (Fe)	0.20		
Phosphorus (P)	0.05		
Iron (Fe)	1.85		

**Table 4:** Thermal data of PPW/SBSBC, PPW/SISBC, PPW/SBSBC/n-Clay, and PPW/SISBC/n-Clay samples obtained from the first heating and cooling process.

Sample	ΔH_m1_ (J g^−1^)	T_m1_ (°C)	ΔH_m2_ (J g^−1^)	T_m2_ (°C)	T_c1_ (°C)	T_c2_ (°C)	$\chi_{c}$_1_ (%)	$\chi_{c}$_2_ (%)
PPW	−29.5	127.9	−33.3	164.6	115.6	121.7	10.1	24.1
PPW/SBSBC	−25.5	129.3	−28.3	164.0	114.8	120.4	8.4	19.5
PPW/n-Clay 5%	−24.4	130.8	−30.7	165.7	113.5	120.1	8.3	22.0
PPW/n-Clay 10%	−25.7	129.7	−28.2	165.3	114.7	120.8	8.9	20.4
PPW/SBSBC/n-Clay 5%	−23.7	128.2	−39.9	165.0	114.3	120.0	7.8	27.5
PPW/SBSBC/n-Clay 10%	−22.7	129.5	−25.7	165.2	115.0	120.4	7.1	17.0
PPW/SISBC	−25.6	129.6	−28.5	164.4	114.3	120.6	8.8	20.6
PPW/SISBC/n-Clay 5%	−24.0	128.1	−26.4	165.0	114.8	120.4	7.9	18.2
PPW/SISBC/n-Clay 10%	−19.8	130.7	−31.6	165.5	114.6	120.4	6.2	20.8

**Table 5:** The average crystallite size of PPW composites.

Sample	Average Crystallite Size (nm)
PWP	42.24
PPW/SBSBC	32.58
PPW/SBSBC/n-Clay 5%	26.98
PPW/SBSBC/n-Clay 10%	32.38
PPW/SISBC	25.41
PPW/SISBC/n-Clay 5%	16.12
PPW/SISBC/n-Clay 10%	25.14

**Table 6:** Thermal parameters evaluated from TGA curves.

	Weight Loss (%)	Weight Loss (%)	Tmax_1_ (°C)	Tmax_2_ (°C)	Weight Loss (%)	Tmax_3_ (°C)	Residue (%)	Temp. (°C)	Weight Loss (%)
	25–310 °C	310–560 °C			560–700 °C		700 °C (N_2_)	Onset degradation	
n-Clay	1.90	6.48	488.6	-	1.21	-	90.26	431.0	97.91
PPW	1.96	83.42	463.2	-	5.01	631.5	9.58	446.5	96.04
SBSBC	0.25	99.24	452.4	-	0.22	-	0.21	415.5	99.63
PPW/SBSBC	1.58	85.15	461.9	-	4.50	641.9	8.73	435.0	96.73
PPW/n-Clay 5%	1.37	79.07	463.5	-	4.63	645.8	14.93	444.4	96.87
PPW/n-Clay 10%	1.26	77.49	463.6	-	4.59	649.2	16.66	444.3	97.04
PPW/SBSBC/n-Clay 5%	1.21	81.42	462.4	-	4.33	652.3	13.04	435.2	97.14
PPW/SBSBC/n-Clay 10%	1.25	78.11	463.0	-	4.19	647.5	16.44	434.2	97.20
SISBC	0.92	98.61	375.2	423.7	0.03	-	0.45	351.0	96.85
PWP/SISBC	1.45	85.43	370.7	458.3	4.46	641.4	8.65	429.4	95.95
PPW/SISBC/n-Clay 5%	1.28	81.15	376.7	458.7	4.27	649.7	13.31	428.9	96.26
PPW/SISBC/n-Clay 10%	1.26	78.30	375.5	458.9	4.09	651.1	16.35	428.8	96.42

**Table 7:** Mechanical characteristics for PPW/SBSBC, PPW/SISBC, PPW/SBSBC/n-Clay, and PPW/SISBC/n-Clay composites compared with those for PPW and PPW/n-Clay.

Sample	Tensile Strength at Break (MPa)	Elongation at Break (%)	Hardness (Sh D)	VST A50 (°C)	HDT (°C)	Izod Impact (kJ m^−2^)
PPW	24.12 ± 2.4	28.30 ± 3.1	63.5 ± 1	138 ± 2	168 ± 3	1.35 ± 0.5
PPW/SBSBC	19.62 ± 1.2	64.32 ± 4.5	61.5 ± 1	136 ± 2	167 ± 2	2.65 ± 0.3
PPW/n-Clay 5%	22.78 ± 2.0	25.00 ± 5.0	62.0 ± 1	130 ± 2	165 ± 2	1.15 ± 0.4
PPW/n-Clay 10%	22.56 ± 3.1	15.92 ± 2.4	62.5 ± 1	132 ± 2	166 ± 2	1.10 ± 0.2
PPW/SBSBC/n-Clay 5%	19.45 ± 0.9	35.22 ± 4.7	61.5 ± 2	110 ± 1	165 ± 2	2.19 ± 0.2
PPW/SBSBC/n-Clay 10%	18.25 ± 1.2	31.30 ± 4.4	62.0 ± 2	113 ± 2	163 ± 5	1.78 ± 0.3
PPW/SISBC	20.12 ± 1.9	70.14 ± 3.7	60.5 ± 1	135 ± 2	166 ± 5	3.89 ± 0.7
PPW/SISBC/n-Clay 5%	19.62 ± 0.5	64.32 ± 4.7	61.5 ± 1	136 ± 2	167 ± 4	3.25 ± 1.0
PPW/SISBC/n-Clay 10%	22.78 ± 0.7	35.40 ± 6.0	62.0 ± 2	130 ± 1	165 ± 6	2.15 ± 0.6
